# A Comparison of Plasmid DNA and mRNA as Vaccine Technologies

**DOI:** 10.3390/vaccines7020037

**Published:** 2019-04-24

**Authors:** Margaret A. Liu

**Affiliations:** ProTherImmune, 3656 Happy Valley Road, Lafayette, CA 94549, USA; Liu@ProTherImmune.com

**Keywords:** DNA vaccine, mRNA vaccine, plasmid DNA, in vitro transcribed mRNA, immune responses, formulations, Cytolytic T Lymphocytes, antibodies, innate immunity

## Abstract

This review provides a comparison of the theoretical issues and experimental findings for plasmid DNA and mRNA vaccine technologies. While both have been under development since the 1990s, in recent years, significant excitement has turned to mRNA despite the licensure of several veterinary DNA vaccines. Both have required efforts to increase their potency either via manipulating the plasmid DNA and the mRNA directly or through the addition of adjuvants or immunomodulators as well as delivery systems and formulations. The greater inherent inflammatory nature of the mRNA vaccines is discussed for both its potential immunological utility for vaccines and for the potential toxicity. The status of the clinical trials of mRNA vaccines is described along with a comparison to DNA vaccines, specifically the immunogenicity of both licensed veterinary DNA vaccines and select DNA vaccine candidates in human clinical trials.

## 1. Introduction 

Plasmid DNA [1] and now mRNA [2] vaccines have generated significant interest and efforts because of their potential as platform technologies that could be used for a variety of applications ranging from prophylaxis to therapy and from personalized medicine to global health solutions. Both can be quickly made with fairly generic manufacturing processes and can be constructed directly from the genetic sequence of the desired protein, whether the origin of the protein is human or from a pathogen. For vaccines, making a gene construct coding for the antigen instead of inactivating or attenuating the pathogen, or instead of making a recombinant protein, is vastly easier, more rapid, and avoids potential risks of working with live pathogens. Likewise, the vaccine construct can encode only the key antigen without including other proteins that may be either deleterious (such as toxins) or that may be irrelevant for protection yet immunodominant. 

The ease and speed of making the constructs also means that these are considered potential gamechangers for targeting epidemic or emerging diseases where rapidly designing, constructing, and manufacturing the vaccine are crucial. For cancer, rather than relying on tumor-associated antigens that are common to many tumors, it would require little more effort to make the vaccines specific for that individual’s exact tumor antigens, now referred to as personalized vaccines. The concept was demonstrated pre-clinically in the mid-1990s with DNA vaccines targeting lymphoma, where the idiotype of a tumor could be rapidly sequenced, and a DNA vaccine made much more quickly than a recombinant protein version [3,4]. Alternatively, as is being tested now for mRNA [2,5], libraries of gene-based constructs encoding various antigens could be made. Then, based on a patient’s individual tumor antigens, a combination of constructs could be easily combined from this pre-made library.

At a time when many scientists are turning from plasmid DNA to explore mRNA technology while remaining uncertain about when or whether DNA vaccines will be licensed for human diseases, it is useful to compare the two technologies by analyzing both the theoretical issues and the experimental data and progress for both.

## 2. Background

In 1990, Felgner and colleagues published, in Science [6], their demonstration that so-called “naked DNA”, that is, plasmid DNA that was not formulated in transfecting agents, could be directly injected into muscle with resultant expression of the encoded protein by myocytes. The observation was important because up until then, significant effort had been devoted to formulations to deliver DNA in vivo, and many such compounds were used for in vitro transfection. The surprising simplicity of the approach generated significant interest, and when it was soon shown (in 1993) that plasmid DNA coding for a conserved internal influenza protein could generate protection in a pre-clinical mouse model against influenza challenge with a very different influenza strain than the strain from which the protein antigen was sequenced [7], many groups began developing plasmid DNA for vaccines, cancer immunotherapies, and immune interventions for autoimmune and allergic diseases.

The same 1990 publication also demonstrated that naked RNA could similarly result in the in vivo expression of encoded protein. However, more attention focused on utilizing plasmid DNA, rather than mRNA, likely because of concerns about the instability of mRNA. In 1992, Bloom and colleagues [8] demonstrated the efficacy of mRNA to express protein in vivo by showing that mRNA encoding a hormone could correct a disease following direct injection into rat brains. In the same year (1993) that the first demonstration of the ability of DNA plasmid to protect mice from heterosubtypic challenge with influenza was published [7], liposome-formulated mRNA was also shown to generate influenza-specific cytolytic T cells in mice [9] (although protection from infectious challenge, as was shown for plasmid DNA, was not tested, perhaps explaining part of the difference in excitement about the technologies). Nevertheless, for both entities, a key issue was how to optimally deliver the DNA plasmid or the mRNA into the desired cells, either for optimal expression of the desired therapeutic protein as a drug or for gene therapy (to supply a missing or defective protein), or to generate the desired immune response against the protein if it were an antigen. For gene therapy, the encoded protein needs to not stimulate an immune response. For a vaccine, which cell produces the protein encoded by the mRNA or the plasmid DNA can be a key issue because, although for antibodies [10] where the protein would likely need to be secreted, for cellular immune responses of the Cytolytic T Lymphocyte variety [11], the type of cell producing the protein (and hence the cell type transduced by the plasmid DNA or the mRNA) is relevant, as is discussed later.

Why was there relatively less interest in mRNA compared to plasmid DNA as a platform technology for over a decade? What led to the recent explosion of interest and progress for mRNA? The transient nature of messenger RNA, possibly an asset for the process whereby organisms control the production of desired proteins, is due to RNAses that are widely present [12]. This instability of mRNA has been a significant reason for the lack of interest in mRNA as a drug. In addition, RNA has long been known to be an immunologically active molecule. For example, poly (I:C) (polyinosine-polycytidylic acid) is a synthetic analog of dsRNA (double-stranded RNA) that is an agonist of TLR3 and has long been used as an immunostimulatory mimic of viral infection and tested as an adjuvant to increase immune responses for experimental vaccines [13,14,15]. mRNA has a number of immunostimulatory mechanisms, which may be useful—or detrimental—for mRNA used for vaccines or cancer immunotherapeutics (discussed below). However, these properties contributed to the lower degree of interest in mRNA versus plasmid DNA for gene therapy applications when provision of a missing or defective protein with no immune responses against either the protein or the vector delivery system was the goal. 

Two developments were important for changing the perception and reality of mRNA. These were the demonstration by Weissman and Kariko that the use of modified nucleosides made in vitro-transcribed mRNA less immunogenic [16]. In follow-up work, they showed that using pseudouridine instead of uridine resulted in mRNA that was more stable and had increased translational capacity [17]. This use of modified nucleosides thus addressed key issues for mRNA—stability of the mRNA, increased production of the encoded protein, and some decrease of the innate immunogenicity. Additional work explored the use of other nucleosides, such as substituting 5-methylcytidine for cytidine with further improvement [2,11].

## 3. mRNA Structure and Implications for Use as a Vaccine

At this stage, it is perhaps useful to review the structure of the mRNA as designed for drug and vaccine delivery, which incorporates elements to improve both stability and protein expression. The mRNA comprises a 5′ cap, a 5′ untranslated region (UTR) (also called leader RNA), the coding sequence with a stop signal, a 3′ UTR, and a poly(A) tail. This molecule provides the template in the cytoplasm of a cell for translation by the ribosome and tRNA into the encoded protein, making multiple copies of the protein from each mRNA template. This amplification provides a quantitative advantage per molecule compared to providing individual proteins. However, offsetting that numeric advantage is that, in addition to the instability of the mRNA, it is thought that only one out of 10,000 molecules of mRNA will escape an endosome into the cytoplasm [5]. The amplification by translation of the mRNA into protein has to overcome the losses and the inefficiencies of degradation and the transduction process. Another obvious implication for this is that, compared to plasmid DNA, which must enter the nucleus of a cell, the mRNA only needs to be present in the cytoplasm, which eliminates the additional cellular (i.e., nuclear) membrane that plasmid DNA needs to cross. On the other hand, plasmid DNA is more stable than mRNA, and each DNA molecule results in the production of multiple mRNA molecules, thus the theoretical advantages of one over the other boil down to the realities of the net stability of plasmid DNA versus mRNA in their final formulation, as well as the efficiencies of targeting to the desired cell, the transduction to the cytoplasm or nucleus followed by the efficiencies of transcription of the plasmid DNA (resulting in amplification from DNA to mRNA), and the translation of mRNA, whether transcribed from DNA or in vitro-transcribed mRNA, to protein (also resulting in amplification).

Lower quantities of the (antigenic) protein are presumably needed for vaccines (due to amplification of the immune response against the antigen) compared to amounts of protein that might be needed for therapeutic disease targets. Additionally, whereas for gene therapy, where long-lasting or even permanent production of the therapeutic protein is desired, vaccines likely benefit from the transient nature of the antigen (followed by boosting). This is because, for example, the development of high affinity antibodies occurs as antigen becomes scarcer. Subsequent boosts with antigen then expand the production of these high affinity antibodies. The relatively temporary nature and presumably small amounts of protein produced by mRNA would fit with this paradigm if the mRNA is present in great enough quantities, persists, and is active long enough to produce sufficient amounts of protein antigen to stimulate the desired immune responses. DNA vaccines likewise have been demonstrated to produce the encoded protein for a limited period of time, although this is likely longer than mRNA constructs given the greater inherent stability of plasmid DNA compared to mRNA. Plasmid DNA has been shown to persist in muscle up to six months in a non-integrated fashion [18].

As noted above, the ability to make either a plasmid DNA or an mRNA construct quickly by simply knowing the genetic sequence of a desired antigen makes plasmid DNA or mRNA much faster technologies (compared to current approaches) to produce a vaccine, if needed, for an epidemic or an emerging disease. Five characteristics—rapidity of making constructs, relatively temporary presence in vivo of the encoded protein, amplification by the immune system responding to even small amounts of expressed protein, the manufacturing advantages (generic and rapid processes compared to drugs or recombinant proteins), and the intrinsic immunostimulatory properties of both plasmid DNA and mRNA—combine to make a compelling rationale for vaccines to be viewed as the best initial targets for widespread development efforts for both technologies.

## 4. Manufacture

The manufacture of plasmid DNA has been considered to be one of its strengths, making it a platform technology where the same process could essentially be used regardless of the gene that was encoded [19]. Moreover, the process of bacterial fermentation is fairly simple, since the product is a plasmid grown in bacteria, such as *Escherichia coli*, and the plasmid DNA is relatively stable, making purification straightforward. This is in contrast to the time-consuming process of earlier generation vaccines, which required finding ways to grow the pathogen such as making it weaker or inactivating it. Historically, the process to develop vaccines, including the manufacturing process, has been long and could reach up to decades (e.g., the chicken pox vaccine). The advent of recombinant proteins provided a simpler means of making vaccine antigens, and one that eliminated the need to work with a virulent pathogen during manufacture. However, this still had drawbacks, such as ensuring that the antigen had any crucial antigenically correct post-translational modifications (such as glycosylations), which can differ between host cells (such as yeast or baculovirus compared to humans), that the antigen was properly folded, and so on. Recombinant proteins generally also need to be soluble, providing a challenge for proteins with a transmembrane domain that is needed either antigenically or for any necessary oligomerization [e.g., HIV envelope]. Recombinant proteins administered exogenously (e.g., given in an immunization) also have an inherent limitation of not stimulating Major Histocompatibility Complex (MHC) Class I-restricted Cytolytic T Lymphocytes (CTLs), as is discussed later.

mRNA is made by in vitro transcription starting from a linearized DNA template, performing in vitro transcription, then getting rid of the template by digestion with DNAses, at which point the mRNA can be purified. Manufacturing mRNA by in vitro transcription is thus even more appealing than manufacturing plasmid DNA because while it is also a generic process, (i.e., independent of the gene insert), it is essentially a chemical process with no animal or cellular components (although the cost is potentially greater [20]). A graphic detailing the various steps and suggested possible improved processes can be seen in the reference [21]. The manufacturing process might be guided by pharmaceutical product Good Manufacturing Practice (GMP) guidelines [22,23] rather than those for biologicals [24], a likely advantage. Of course, any formulations or the addition of immunomodulators, adjuvants, or delivery systems may increase the complexity and cost of the manufacture for either mRNA or plasmid DNA. 

## 5. Stability as a Product

DNA vaccines as a manufactured entity are noted for their stability [25], particularly when supercoiled. As noted above, this stability is reflected even in vivo, since plasmid has been detected in a non-integrated form in muscle up to six months following injection [18]. Although the ubiquity of RNAses with the resulting instability of native mRNA has been a significant reason for the delay in development of mRNA, the actual manufactured mRNA is stable in liquid or lyophilized form, (reported to be stable up to two years at room temperature [26]) with an inverse relationship of stability to temperature. It has been reported that a rabies mRNA vaccine was still effective for pre-clinical protection after several months at temperatures ranging from −80 °C to as high as +70 °C [26]. The stability of mRNA as a vialed product (i.e., protected from RNAses) is a separate consideration from the stability in vivo, and any formulations or delivery devices (which may cause shearing during delivery) are key factors in the final stability as a delivered product. 

## 6. Cellular Targets for mRNA and Plasmid DNA Vaccine Delivery

For many vaccines, antibodies play a key role in protection. The cell that is transfected by the plasmid DNA or the mRNA vaccine does not have to be a professional antigen presenting cell (APC) in order to produce the antigenic protein that stimulates B cells. Cellular immune responses, notably CTLs, are thought to be important for tumor immunotherapy as well as to potentially play a role in protection against certain infectious diseases, e.g., tuberculosis (Tb), HIV, and malaria, or for vaccines effective against multiple strains of a virus, such as influenza, even though CTLs alone would not provide sterilizing immunity. In order for a vaccine to generate MHC Class I-restricted CTLs, the antigen either needs to be produced inside a professional APC or by a cell from which antigen can be cross-presented by an APC to then stimulate CTLs. The most direct way to ensure delivery of the gene encoding an antigen to a professional APC is to transfect the cells in vitro prior to administering the transfected cells back to the patient. Indeed, the largest number of mRNA clinical trials currently underway, notably for cancer immunotherapy, involve the ex vivo transfection of cells with mRNA encoding tumor antigens followed by re-infusion of the transfected cells into the patient. Because this is a more cumbersome process for making a product than having a non-personalized product in a vial, direct administration of the plasmid DNA or the mRNA to a patient is preferable for convenience, cost, and time. 

Plasmid DNA was shown to be effective for stimulating CTLs that were capable of protecting mice against influenza caused by a strain different from the strain from which the encoded antigen was derived [7,27,28]. Because the plasmid DNA, when injected intramuscularly (i.m.), primarily transduced muscle cells rather than professional Antigen Presenting Cells (APCs), the mechanism whereby MHC Class I-restricted CTLs were generated needed explaining. It was found that cross-priming appeared to be a key mechanism for generating CTLs following DNA vaccine immunization, as directly demonstrated by experiments with chimeric mice using bone-marrow derived dendritic cells [27], and because muscle cells were the only cells observed to translate the protein encoded by directly-injected plasmid DNA [6,29]. The efficacy in pre-clinical models raised the hopes that such plasmid DNA-based CTL-inducing vaccines could be developed that would be protective against multiple strains of HIV or influenza [7,30] (so as to produce a “universal” flu vaccine). Currently, existing influenza vaccines depend upon strain-specific antibodies, which result in strain-specific or strain-limited protection. Similarly, mRNA delivered in liposomes was shown early on to be capable of inducing CTLs [9]. The uptake of the mRNA is also mainly by non-immune cells, including muscle cells [31].

Both plasmid DNA and mRNA are also being developed for indications other than vaccines, such as for gene therapy. The delivery of plasmid DNA and mRNA to specific tissues or cells may thus be intentionally directed (in part) by the mode of delivery, the injection route, the formulation, and so on. Both entities are anionic due to the negative charges of the phosphate groups, and various formulations have utilized polycations. Thus, the biodistribution of each molecule depends not simply on the inherent charge and the size of the plasmid or mRNA, but the net charge of all the components of the formulation and the effect of any lipids.

## 7. Increasing the Potency of DNA and mRNA Vaccines

For DNA vaccines, despite the ease with which preclinical studies demonstrated efficacy for a variety of disease models, the potency in humans proved generally disappointing. This led to a number of approaches to increasing the potency by increasing the amount of protein produced through redesigns of the plasmid. Additionally, adjuvants and other immunostimulants were included (such as cytokines and co-stimulatory molecules) either as recombinant proteins or encoded by plasmid DNA, by various formulations and delivery devices, and by strategies such as prime-boost combinations (generally using plasmid DNA as a prime followed by a heterologous boost with a viral vector or protein). The DNA plasmids themselves were optimized by trying different promoters, adding CpG motifs, (cytosine connected via a phosphodiester bond to guanine-such CpG motifs are pathogen-associated molecular patterns) (see below), codon optimization, etc. As noted above, the initial work by Felgner [6] demonstrated that the expression of protein encoded by plasmid DNA was highest in muscle following intramuscular injection versus expression in other tissues after intravenous or subcutaneous injection. Likewise, immune responses were highest with direct i.m. syringe injection of naked plasmid DNA rather than via intravenous (i.v.), intradermal (i.d.), or subcutaneous (s.c.) injections [7]. Early delivery devices for plasmid DNA included a biolistics gene gun that propelled DNA-coated gold particles into cells [32]. In addition to simple i.m. injection, approaches now include pressurized devices (such as the Biojector® or Stratis®), or electroporation, which supplies an electric current to cause temporary fenestration of membranes to increase the passage of plasmid into the cells and the nuclei.

For mRNA, the areas of continued Research and Development (R&D) efforts for improving the potency of mRNA vaccines are shown in Table 1, which describes efforts similar to those for DNA vaccines. These focus on augmenting delivery of the mRNA and increasing potency via increased stability and greater expression of the protein. Alterations of the mRNA itself include changing the codon usage and the GC (guanine-cytosine) content [5], along with modifications of the other regions, such as the 5′ cap, the UTRs, and the poly-A tails. A more detailed description of the efforts to increase the mRNA potency and of delivery formulations that include lipids, nanoparticles, polymers, polycations, and various proprietary entities are presented and reviewed elsewhere with tabular and chemical descriptions [2,11,33,34]. Cells generally take up mRNA by endocytosis, thus efforts are also being made to design delivery systems that increase the endosomal release of the mRNA into the cytoplasm [35]. Certain formulations, such as delivery of a particular encapsulated lipoplex mRNA vaccine, were found to specifically be taken up by dendritic cells via micropinocytosis [36]. As with DNA vaccines, possible immunomodulators added as recombinant proteins or encoded by mRNA are being evaluated [37]. Various routes of injection of mRNA are being explored, including i.m., i.d., s.c., i.v., and intranodal [2], in addition to the ex vivo approach described. Delivery devices such as the gene gun (where mRNA is put onto gold particles) [38] and electroporation are also being explored.

Circular RNAs (circRNA) are endogenously expressed and are thought to play roles mainly for gene regulation, with potential activity as tumor antigens [39]. They can be exogenously constructed to produce proteins in cells [40]. These engineered circRNA molecules appear to be more stable and to result in more potent production of protein than linear mRNA. However, the mechanisms for their effects upon gene regulation and other activities are still being explored [41]. 

### 7.1. Self-Amplifying Systems for Both mRNA and DNA Vaccines 

Significant efforts have been expended to take advantage of a system employed by certain viruses, notably alpha viruses, which utilize a strategy of self-amplification of key viral proteins. Such self-amplifying replicon systems have been developed for viral vectors, plasmid DNA, and now mRNA [42,43,44,45]. These constructs encode viral proteins that result in the transduced cell producing many copies of mRNA encoding the protein of interest (i.e., the antigen) without making a whole viral particle. Thus, for a given DNA or mRNA vector, significantly more mRNA encoding the antigen and hence antigen protein, are made. In pre-clinical models, this has resulted in increased potency for these vectors on a per molecule of vector basis. The reason for the increased efficacy may be more than simply the increased amount of antigen produced, as the dsRNA intermediaries result in increased production of interferon and subsequently other immunologic effects, although the dsRNA can also have other possibly deleterious effects (see below). 

## 8. Inflammatory Responses and Toxicities

### 8.1. Immune Activation

While both DNA and mRNA vaccines are often thought of as simply an expression system for the desired protein, neither is immunologically inert. Both DNA vectors (which are based on bacterial plasmids) and in vitro transcribed mRNA activate the innate immune system. DNA plasmids do so via their CpG motifs, which stimulate TLR9. While CpG was successfully used as an adjuvant [46] for a recombinant protein-based Hepatitis B vaccine licensed in 2018, the impact on the immunogenicity of DNA vaccines by increasing the number of CpG motifs in the plasmid has been less clear. In fact, for certain DNA vaccine efforts, notably those of Steinman’s group, as therapies for autoimmune diseases, they specifically switched CpG motifs for GpG motifs (guanine connected via a phosphodiester bond to another guanine; these compete with CpG motifs for binding to TLR9 receptors) in an effort to specifically decrease the Th1 help for their human clinical studies (see below) [47]. The double-stranded structure of the DNA plasmid is also thought to be an immune stimulant [48] through non-TLR mechanisms. In fact, plasmid DNA also acts on the TBK1-STING pathway through cytosolic receptors [49,50]. This results in the generation of Type 1 interferons, which then act as adjuvants for the generation of immune responses against the antigen(s) encoded by the plasmid DNA vaccine.

As noted above, the use of modified nucleosides for the construction of mRNA is one method of decreasing the reactogenicity of the in vitro transcribed mRNA. However, mRNA acts via multiple pathways, including the innate system (via TLR3, TLR 7, and TLR8) and via cytoplasmic proteins (PKR, OAS, RIG-I, and MDA5) [11,51]. The multiple routes of activation result in several effects in addition to inflammation and include inhibition of mRNA replication (both via TLR7 through an MYD88 pathway affecting interferon, and via TLR3 through TRIF), stalled translation, and RNA degradation [5]. Some of these various activities could decrease the potency of the mRNA by a net decreased protein production, as was seen pre-clinically for an HIV mRNA vaccine complexed in cationic lipids [52]. This also raises the issue of how effective repeat dosing of mRNA will be if previous injections result in an environment with decreased translation or increased RNA degradation, although simply changing an injection site may potentially circumvent this particular issue.

Other molecular entities that are introduced or generated during the manufacture of the in vitro-transcribed mRNA and then remain (left-over contaminating nucleoside triphosphates, DNA templates, and dsRNA) are also quite immunostimulatory and therefore need to be purified following production of the mRNA [53].

The potential issues due to the various inflammatory effects of mRNA vaccines upon clinical efficacy and safety are summarized in Table 2 and are discussed below. The possible utility of RNA-induced inflammation for vaccines is demonstrated by the fact that one of the first uses of RNA for vaccines was to include non-coding RNA in human clinical trials as an adjuvant for a rabies vaccine (composed of an inactivated virus) [54], although this effort has been replaced by a rabies vaccine that utilizes mRNA that itself encodes the rabies antigen [55], as is discussed below. The continued evaluation of non-coding RNA as an adjuvant is ongoing in clinical testing for various cancers without the provision of an antigen (see below).

### 8.2. Toxicities of mRNA

The flip side of the possibly beneficial adjuvant inflammation, however, is potential toxicity of the mRNA vaccines. Toxicities are seen with antivirals and anti-cancer drugs that contain unnatural nucleoside analogues [56,57,58]. Such toxicities, not predicted by pre-clinical studies due to species differences between humans and the animals used for pre-clinical safety testing, have been seen with drugs that contain unnatural modified nucleosides. The clinical adverse effects have included myopathy (caused by mitochondrial toxicity), lactic acidosis, pancreatitis, lipodystrophy, liver steatosis, and nerve damage; certain ones have been fatal.

Indeed, some toxicity has been reported for mRNA pre-clinically along with limited human adverse events. Liver toxicity was observed in pre-clinical studies with one potential mRNA therapeutic delivered in lipid nanoparticles for Crigler-Najjar syndrome, selected as a “lowest-hanging fruit” target because very low doses of protein were needed. These were serious enough to apparently halt the work with this particular entity, or at least that formulation [59]. The formulation of the mRNA was thought to potentially play a role in the toxicity [60], and repeat doses were used. Nevertheless, this observed toxicity may be concerning for vaccines as well, since even live replicating viruses and viral vector vaccines (which generally are more immunogenic than subunit vaccines) need repeat dosing. In addition, most of the mRNA vaccines in clinical trials appear to need formulation. The mRNA vaccines in clinical trials against infectious diseases from this same company are described as formulated in lipid nanoparticles, but whether they are the same formulations as those used for the Crigler-Najjar study is not publicly known.

Self-limited local and systemic adverse events (AEs) seen in a human clinical trial for an mRNA rabies vaccine, although summarized as still indicating the vaccine was generally safe (described below in the clinical trials section), may also reflect the inflammatory nature of the mRNA [55]. These results highlight the potential toxicity downside of the inflammatory activity of mRNA vaccines, adverse effects not seen to this extent with plasmid DNA. Also note that, for providing monoclonal antibodies [61] (whether for preventing or for treating infectious diseases for other therapeutic applications), this would likely require repeat administration of mRNA, which might not only increase the potential for toxicities, but may also have an impact upon potency due to effects of the mRNA upon decreasing translation, etc., via the other inflammatory effects.

Thus, it may still be a work in progress to find the best balance of inflammation and any deleterious toxicities via harnessing adjuvant activities of mRNA while limiting or suppressing inherent toxicities for vaccines and immunotherapeutics. This will involve optimizing nucleoside substitutions, the design of other elements of the mRNA construct, any included immunostimulants, and/or specific formulations, delivery devices, and routes of administration. The mechanisms of mRNA inflammation that are relevant to their potential efficacy and safety as vaccines are also reviewed elsewhere [34], where they are aptly referred to as the “yin and yang of innate immunity”.

## 9. Other Potential Safety Issues

When DNA vaccines initially entered into human clinical trials, concern was raised about the theoretical possibility of them causing autoimmunity or that the DNA would integrate into the genome. The rationale for concerns about autoimmunity was that anti-DNA antibodies are a hallmark of various autoimmune diseases. To date, both pre-clinical testing and careful clinical monitoring have shown DNA vaccines to not induce or to worsen auto-immunity, and in fact, human clinical trials employing DNA vaccines for therapy of two autoimmune diseases (diabetes mellitus and multiple sclerosis) gave encouraging results in human clinical trials for a therapeutic benefit of the DNA vaccines [47,62]. For mRNA, a proposed mechanism for possible autoimmune responses is via the induction of type I interferon [63], which may result in both inflammation and possibly autoimmune responses [64]. This includes work showing that the responses seen in mice were similar to those seen in humans for an influenza mRNA vaccine construct via TLR7 and TLR8 in humans and via cytoplasmic RNA sensors in both mice and humans [65].

DNA vaccines did not need to be evaluated by the US National Institutes of Health (NIH) Recombinant Advisory Committee prior to human clinical trials, unlike viral vectors for gene therapy. Nevertheless, significant safety studies were initially required to evaluate the possibility of integration of the plasmid DNA into the host genome. As a result of these studies for both human vaccines [18,66] and for the licensed DNA vaccines for fish [67], as well as the many human studies with DNA vaccines that have demonstrated safety, little concern now exists regarding integration. Comparisons have stated that mRNA offers an advantage because RNA itself cannot integrate into genomic DNA without the presence of the viral elements in a retrovirus that enable such integration (reverse transcriptase and integrase). However, HERVs [68] (human endogenous retroviruses) whose remnants are now permanent parts of human genomes as retrovirus-like sequences comprise up to 8% of the human genome. In addition, some recipients of mRNA drugs or vaccines may be already infected with a retrovirus (e.g., HIV), thus providing a theoretical means for provision of the proteins needed for integration [69,70]. Nevertheless, the risk of integration remains, at this point, extremely unlikely for mRNA, even from a theoretical standpoint, nor is it any longer a significant concern for plasmid DNA. This means that mRNA does not offer any clear advantage compared to plasmid DNA in this regard. From a regulatory perspective, mRNA prophylactic vaccines appear to not be considered gene therapy products [71], similar to DNA vaccines before them.

## 10. Clinical Trials

### 10.1. DNA

#### 10.1.1. Licensed Veterinary DNA Vaccines

Five plasmid DNA products (four of them vaccines) have received licensure for veterinary applications. These include a fish vaccine for infectious hematopoietic necrosis virus licensed in 2005, and a vaccine against salmon pancreas disease licensed in 2016 [72]. A dog cancer immunotherapeutic vaccine for melanoma was licensed originally based upon comparison with historic controls in the US in 2010. After submission to the European Medicines Agency, the application was withdrawn in 2014 by the company, stating that their priorities had changed such that they did not justify the investment in research and development to answer the remaining questions [73]. A vaccine for West Nile virus (WNV) prevention in horses was licensed in 2005 [74], although it is no longer used in favor of the previously licensed killed virus vaccine for unpublished reasons. Yet, a promising observation was that the equine WNV DNA vaccine was able to protect various species of birds from WNV [75,76,77,78], including the California condor, and has been credited by U.S. Center for Disease Control (CDC) scientists with saving this endangered species from potential extinction [79]. A fifth plasmid DNA product was licensed in 2008 for veterinary use; this plasmid encodes growth hormone releasing hormone (GHRH) and is given via electroporation to pregnant sows, resulting in litters with an increased number of surviving piglets and of higher birthweights [80,81].

#### 10.1.2. Significance of Licensed Veterinary DNA Vaccines for Human DNA Vaccines

The licensure and the immunogenicity of the equine WNV vaccine are significant for human DNA vaccine efforts. The first reason is that scientists have often stated that DNA vaccines are not very good at inducing antibodies, yet this DNA vaccine induced neutralizing antibodies of sufficient titer for protection and licensure in horses. Also significant is that these antibodies were made in horses. Frequently, the lack of potency of DNA vaccines in human trials was considered to reflect the size of humans compared to the usual small pre-clinical animal models.

#### 10.1.3. Select Human DNA Vaccine Clinical Trials Results

In related observations to the equine WNV DNA vaccine, in a human clinical trial of a WNV DNA vaccine in humans, all subjects generated titers of antibodies that were considered protective in the horses [82]. In a subsequent study using a construct with a stronger promoter, older adults (who generally are considered to have senescent immune systems and respond to licensed vaccines such as the influenza vaccine more poorly than younger persons) had neutralizing antibody responses as good as the younger adults [83]. In a clinical trial of a DNA vaccine for Ebola and Marburg viruses, the individuals likewise generated antibodies that were boostable [84]. These observations demonstrate that DNA vaccines are capable of inducing antibodies in humans of relevant titers, suggesting that it is not a limitation of the technology per se to generate effective antibodies, but rather the target and the optimized constructs are key elements (much as finding the right target for monoclonal antibodies (MAbs) was needed for MAbs to become such effective anti-cancer agents). Also of note is that the first two Zika vaccines brought to human clinical trial were DNA vaccines [85]. This underscores the point made earlier about how the ease of making both DNA and mRNA vaccines is considered a tremendous advantage for rapid responses to emergent or epidemic diseases.

#### 10.1.4. Additional Categories of Disease Targets for DNA Vaccines and Methods to Increase Efficacy

In addition to the various diseases for which DNA vaccines used alone have resulted in promising human clinical immune responses, in a variety of clinical trials for several other diseases, such as HIV, plasmid DNA as a prime followed by a heterologous boost has resulted in significant potency for the generation of immune responses, including CTLs. Additionally, as mentioned above, phase II studies for the treatment of two autoimmune diseases, diabetes and multiple sclerosis, yielded encouraging clinical responses, which may mechanistically be due in part to the design of the vectors to avoid the Th1 cell responses that generally are seen with the plasmids utilized for other diseases [47]. Moreover, clinical trials of DNA vaccines for cancer therapy utilizing electroporation have provided encouraging results, including for CIN3 (cervical carcinoma in situ 3) [86] and CIN2/3 [87] as well as for head and neck cancer [88], which are all related to human papillomavirus (HPV) infection. Other clinical trials of DNA vaccines for cancer have included using DNA that encoded fusion proteins including a tumor CTL epitope(s) and a T helper stimulator(s) [89,90,91]. Therefore, even though no human DNA vaccines have been licensed, the existing data have provided evidence for immunogenicity and early stage evidence of clinical effect in humans for certain antigens/diseases as well as efficacy in animals ranging from fish to horses. The remainder of this special issue deals with these efforts and ongoing clinical trials, thus they are not elaborated upon here.

### 10.2. RNA

#### 10.2.1. Prophylactic mRNA Vaccines for Infectious Diseases

Prophylactic vaccine human trials for infectious diseases utilizing mRNA encoding the antigen(s) are shown in Table 3. These are all Phase I trials. Any known formulations are listed, as are any described results and references, along with the clinical trials identifier numbers. The rabies vaccine effort utilizing a licensed vaccine with RNA as the adjuvant (discussed above, and listed in Table 4) was replaced by a vaccine using mRNA encoding the rabies virus glycoprotein. Following either i.d. or i.m. injection of this rabies mRNA vaccine, boostable antibodies were obtained. However, 78% of each group had “solicited systemic adverse events” including ten patients (~10% of all injected patients) with grade three (i.e., serious but not life-threatening) adverse events, although the conclusion was that the vaccine was “generally safe with a reasonable tolerability profile” [55]. A second construct for rabies is now in clinical testing.

As noted earlier, one company initially highlighted a focus on therapeutic disease areas but reprioritized vaccines, possibly due to the recognition of the low amount of antigen actually produced by the mRNA coupled with the low amounts of protein needed for vaccines because of the amplification by the immune system, possibly as a response to emerging diseases such as the Zika epidemic, and possibly influenced by the liver toxicity seen in their pre-clinical studies [59] for delivering even the low amounts of mRNA needed for the disease (Crigler-Najjar, see above). Lipid nanoparticles are also used for that company’s vaccine formulations, although it is not known how these relate to the formulation used for Crigler-Najjar (the company has published relatively little in the peer-reviewed scientific literature related to their clinical trials). References, when available, are provided within the table, although in some cases, the assumption is made that the pre-clinical construct in the reference is the same construct (antigen) as the one in the clinical trial. 

#### 10.2.2. Additional Clinical Trials of RNA

Table 4 lists clinical trials of mRNA for additional applications. Data are taken from clinicaltrials.gov in addition to the citations listed. Table 4 excludes studies that employ cells transfected ex vivo prior to re-administration to patients and excludes those that supply mRNA encoding immunomodulators without a specific antigen also being provided.

##### RNA as an Adjuvant

Trials utilizing non-coding RNA as an adjuvant are listed. These studies include using non-coding RNA as an adjuvant for a licensed rabies vaccine resulting in improved immunogenicity with mainly mild AEs, but with two of the 14 patients having severe but limited influenza-like symptoms [54]. Studies are ongoing for using this non-coding RNA adjuvant for cancer applications (not using any co-administered mRNA-encoded antigen).

##### Immunotherapeutic Vaccine

mRNA as a therapy for HIV infection was tested in two clinical trials where it was administered intranodally. Although immunogenicity in the Phase I trial appeared promising [92], the second, a phase 2 trial, was terminated after the interim analysis due to lack of immunogenicity above that seen with the placebo. 

##### Immunoprophylaxis via Provision of mRNA Encoding a Monoclonal Antibody

One company recently announced in a press release [93] (no citations found on PubMed for the entity) that a clinical trial has been initiated with mRNA encoding a monoclonal antibody for use in prevention of Chikungunya virus infection.

##### Cancer

For cancer therapy, four completed studies (two each per different mRNA constructs) have been terminated or completed for prostate cancer. Those failed to demonstrate efficacy [98,99], but new trials, including those for other cancers, have been initiated by a variety of groups with various mRNA constructs. Another approach being developed is steering towards personalized cancer immunotherapeutic vaccine products via a library of mRNAs coding for different antigens that can be combined to be personalized for an individual.

As mentioned earlier, a much larger number of trials for cancer utilize mRNA for ex vivo transfection of dendritic cells that are then re-infused into the patient. These are reviewed elsewhere [2,11].

## 11. Summary and Conclusions

In summary, despite all the excitement over pre-clinical efficacy of mRNA, it should be remembered that in many ways, the mRNA field is recapitulating what occurred with plasmid DNA 20+ years ago, when seemingly almost any disease could be prevented or treated in pre-clinical animal disease models with the administration of an unformulated plasmid encoding a key antigen [1]. Therefore, one must keep in mind that pre-clinical immunogenicity or even protection/therapy, and human immunogenicity are low hurdles and are not predictive of human efficacy. One reason this is so challenging is that, for many of the diseases under evaluation, scientists do not know which immune response or combination of immune responses and which antigen targets are the crucial elements for efficacy; the vaccine technology alone is not the only piece of the puzzle. Table 5 summarizes the main advantages and disadvantages of mRNA vaccines with a comparison to DNA vaccines.

Reported Phase I clinical trial results for mRNA vaccines are encouraging, although only the results of the first rabies mRNA vaccine have been published in the peer reviewed literature. The results for the human Metapneumovirus + Parainfluenza virus 3, and the Respiratory syncytial virus (RSV) phase I studies were announced via press release, thus details are not available. The target for the RSV vaccine mRNA was not publicly disclosed at the time the Phase I study was initiated, and to this date, the study appears to not be listed on clinicaltrials.gov. Whether the immune responses are at sufficient levels or have the types of needed immune responses and the necessary duration to result in protective efficacy is unknown and is not necessarily predicted by the Phase I studies.

One should also not ignore the reported toxicities seen with the rabies mRNA vaccine [55] that included limited systemic AEs for the majority of patients (78%) and even grade three AEs in ~10% of patients following doses of 80–400 μg mRNA via different routes, although the conclusions were that the vaccine was generally safe. It is not known whether the pre-clinical hepatic toxicity that proved to be a “no go” result for a particular Crigler-Najjar mRNA candidate is relevant to the mRNA vaccine studies from the same company, because, despite the low doses used, the doses and mRNA formulation for vaccine studies may be different. This is in comparison to DNA vaccine clinical trials where 4 mg doses of DNA i.m. with boosts have been used in a variety of clinical trials with limited systemic symptoms [83,84,85] while generating good immune responses.

Just as DNA vaccines, after more than 25 years since the first publication of preclinical protective efficacy, are still a work in progress in improving potency and finding the right antigens and targets, there remain challenges for mRNA to become clinical products. For both DNA and mRNA vaccines (and monoclonal antibodies and bi-specific antibodies before them), a simple concept may have a challenging path to reality, and the technology may not be totally generic. mRNA may be even more complex than plasmid DNA because of the modifications (modified nucleosides) plus the formulations needed for stability, delivery, and the need to control the innate immunostimulatory activity of the mRNA. However, it also offers advantages in terms of manufacture that avoids the need for any animal or cellular products. The hope is that once the fundamental key challenges are solved for both plasmid DNA and mRNA, the clinical successes will come rapidly, although that has not occurred for moving from the veterinary licensed products for DNA vaccines into humans, demonstrating how much still needs to be understood, not just about the technologies but about the diseases that are being treated or prevented.

## Figures and Tables

**Table 1:** Continued Research and Development (R&D) Focus for mRNA Vaccines.

Stabilize/protect mRNA Target mRNA to desired cells (e.g., professional antigen presenting cells, APCs) Increase escape of mRNA from endosome Deliver mRNA directly to dendritic cells Increase amount of protein translated Increase duration of protein production (may not be needed for vaccines versus therapeutic protein applications) Optimize immune responses for the antigen (e.g., type of T helper response, subclass of antibody) Decrease or select desired inflammatory effects of mRNA Optimize the above for potency, safety, complexity of formulation, cost of manufacture, product stability

**Table 2:** Issues to be addressed for clinical efficacy and safety of mRNA related to inflammation.

Potency: Impact of mRNA innate immune responses (e.g., induction of interferon alpha which slows translation) Potency: Impact of other drugs (antibiotics and anti-cancer drugs) on mRNA metabolism and extent of translation into proteins. Potential toxicity of mRNA due to inherent mRNA inflammatory activity, use of unnatural modified nucleoside, and the formulations; Several pathways of RNA-induced inflammation: TLR 3, 7, 8, plus cytoplasmic pathways Known toxicities of drugs containing unnatural modified nucleosides Potential mitigation or enhancement due to formulation of the mRNA Formulation itself also apparently can affect immune activation and types of immunity (see below, Crigler-Najjar discussion) Will anti-self RNA antibodies be generated and play any role in autoimmune diseases? Design of clinical trials to detect inflammation/toxicity due to mRNA

**Table 3:** Clinical trials for mRNA prophylactic vaccines for infectious diseases.

Product, Company/Institution	Indication (disease)	Antigen	Formulation	Phase	Status	Results	National Clinical Trial Identifier
RNActive® CureVac	Rabies	Rabies virus glycoprotein [55]	None	1	Active, Not Recruiting	Generally safe, but some significant adverse events (AEs); boostable functional antibodies	NCT02241135
RNActive® CureVac	Rabies	Rabies virus glycoprotein	None	1	Recruiting	New construct versus prior trial	NCT03713086
mRNA-1851 Moderna	Influenza H7N9	Influenza Hemagglutinin H7N9 A/Anhui/1/2013 [94]	Lipid Nano-particles	1	Active, Not Recruiting	Moderna website says 1° and 2° endpoints met, but no published data	NCT03345043
mRNA-1440 Moderna	Influenza H10N8	Influenza Hemagglutinin H10N8 (A/Jiangxi-Donghu/346/2013) [94]	Lipid Nano-particles	1	Active, Not Recruiting	Interim: AEs: Majority mild moderate; A few: severe; Seroconversion rates high	NCT03076385
mRNA-1653 Moderna	Human Metapneumo-virus + Parainfluenza virus 3	Fusion proteins of each virus	Lipid Nano-particle	1	Active, Not Recruiting	Announced via press release safe and immunogenic; no publications found	NCT03392389
mRNA-1388 Moderna/DARPA	Chikungunya	Not Disclosed (ND)	ND	1	Active, Not Recruiting	Primary Completion: March 2019; no results posted at time of publication	NCT03325075
RNA-1325 Moderna/BARDA	Zika	prM and E [95,96]	Lipid Nano-particles	1	Active, Not Recruiting	Primary Completion: February 2019; no results posted at time of publication	NCT03014089
mRNA-1647 and mRNA-1443 Moderna	Cytomegalovirus	mRNA-1647 is gB, pentameric complex, and mRNA-1443 is pp65 [97]	Lipid Nano-particles	1	Recruiting	Primary Completion: February 2020	NCT03382405
mRNA-1777 Moderna/Merck-V171	Respiratory Syncytial Virus	ND	ND	1	ND	Moderna press release says 1° and 2° endpoints met, but no published data	Not listed on clinicaltrials.gov

**Table 4:** Clinical Phase I and II trials of mRNA excluding prophylactic infectious diseases (see Table 3) and ex vivo-transduced cells. Information is taken from https://clinicaltrials.gov.

RNA-based Adjuvant: long-chain non-coding RNA complexed with a short cationic peptide (ssRNA adjuvant); no mRNA-encoded antigen Rabies: Phase 1, Completed, ssRNA adjuvant plus licensed rabies vaccine; NCT02238756 [54] Melanoma, squamous cell carcinoma of the skin, squamous cell carcinoma of the head and neck, or adenoid cystic carcinoma: Phase 1, Recruiting, ssRNA adjuvant plus anti-PD-1 therapy; NCT03291002 Hepatocellular carcinoma (HCC): Phase 1/2, Recruiting, ssRNA adjuvant plus multi-peptide-based HCC vaccine; NCT03203005 Therapeutic mRNA Vaccines for Infectious Disease Targets HIV-therapeutic: Phase 1, Completed; NCT02413645 [92] Phase 2, Terminated due to no immunogenicity above placebo at interim analysis; NCT02888756 mRNA Monoclonal Antibody Prophylaxis for Infectious Disease Targets Chikungunya: Monoclonal antibody prophylaxis, Phase 1, Recruiting; NCT03829384 mRNA Vaccines for Cancer (excluding studies where cells are transfected ex vivo, and excluding when no antigen-encoding mRNA is given); multiple groups/companies are sponsors Prostate [98,99]: multiple Solid tumors: including personalized tumor-associated antigens Melanoma and epithelial tumors: multiple trials, personalized, tumor-derived antigens Gastronintestinal cancers Non-small cell lung cancer Breast cancer Various personalized tumor vaccines

**Table 5:** Advantages and Disadvantages of mRNA Vaccines (and comparison to DNA vaccines).

Advantages: 1) Rapid vaccine construction (as with DNA vaccines) 2) Generic manufacturing process (as with DNA vaccines) 3) Manufacturing does not require cells or animal substrates (an advantage from a regulatory perspective compared to DNA vaccines) 4) mRNA does not need to enter the nucleus (an advantage compared to DNA vaccines) 5) Amplification—number of protein antigen molecules produced per molecule of mRNA delivered, compared to no expansion of antigen for traditional antigens (proteins, inactivated virus particles), however, less amplification per molecule of plasmid DNA in the nucleus and likely less amplification than live virus vaccines) 6) Immunostimulatory effects may benefit desired vaccine responses (plasmid DNA also has immunostimulatory effects, but fewer and better defined) 7) Theoretically should not integrate if no endogenous retroviruses or retroviruses due to infection are present. (DNA vaccines have been extensively studied pre-clinically and clinically, easing regulatory concerns about integration for DNA vaccines.) Disadvantages: 1) Amplification to protein antigen per molecule of mRNA is less than that per molecule of plasmid DNA (although the entry into the cytosol is one membrane fewer than needs to be traversed for plasmid DNA) 2) mRNA needs to escape the endosome (but does not need entry into the nucleus, whereas plasmid DNA does) 3) Immunostimulatory effects may decrease potency via multiple pathways: Decreased stability of mRNA Decreased translation into protein Effects upon desired type of immunity 4) Formulation may still be needed (this observation is based upon the use of formulations by the majority of mRNA entities in clinical trials) Finding the optimal delivery formulation/device for humans may be challenging given the unknown predictability of animal models (as with DNA vaccines, although DNA vaccines are much further advanced in clinical trials with different formulations and delivery devices for a number of different diseases) 5) Known toxicity of RNA-based drugs using unnatural modified nucleoside analogues; will this occur with mRNA vaccines? 6) In vitro-transcribed mRNA vaccines may be expensive based on current processes 7) Concomitant administration of other drugs may impact mRNA metabolism and thus may decrease potency of mRNA vaccine
